# Commentary: Efficacy of estradiol–dydrogesterone and auto-cross-linked hyaluronan gel in preventing intrauterine adhesions following missed miscarriage curettage: a retrospective observational study

**DOI:** 10.3389/frph.2026.1797370

**Published:** 2026-02-23

**Authors:** Erkan Mavigök

**Affiliations:** Department of Obstetrics and Gynecology, Faculty of Medicine, Kahramanmaraş Sütçü İmam University, Kahramanmaraş, Türkiye

**Keywords:** Asherman syndrome, hyaluronic acid gel, intrauterine adhesions, missed miscarriage, selection bias

## Introduction

I read with great interest the recent study by Sheng et al. (1), which evaluated the efficacy of combining estradiol–dydrogesterone with auto-cross-linked hyaluronan gel (ACP) to prevent intrauterine adhesions (IUAs) following curettage for missed miscarriage. The authors reported that the combined application of these agents significantly reduced the incidence of IUA and improved menstrual recovery compared to the control group. Given the high recurrence rate of IUA and its detrimental impact on future fertility, exploring multimodal preventive strategies is of paramount importance in gynecological practice.

However, while the findings are encouraging, I believe that the interpretation of the study results requires a more cautious approach due to inherent limitations in the study design. In this commentary, I discuss the potential impact of selection bias and the definition of “standard care” on the reported efficacy of the intervention.

## Selection bias and the lack of propensity score matching

The primary concern regarding the validity of the study conclusions stems from its retrospective observational design and the absence of propensity score matching. In non-randomized clinical settings, the allocation of patients to an intervention group (ACP gel + hormones) vs. a control group is rarely random and is frequently influenced by “indication bias.” Clinicians are more likely to utilize adjuvant preventive measures in patients perceived to be at higher risk for IUA—specifically those with a history of multiple curettages, evidence of infection, or technically challenging surgical procedures involving extensive endometrial trauma.

Conversely, without rigorously adjusting for baseline confounding variables (e.g., duration of surgery, operator experience, or socioeconomic status), it is challenging to attribute the superior outcomes solely to the pharmacological intervention. As noted in methodological literature regarding observational studies, failing to balance these covariates can result in biased estimates of treatment effects (2). If the control group exhibited a higher baseline risk or worse prognostic factors that were not statistically adjusted for, the efficacy of the combined therapy might be overestimated.

## “No-intervention” control vs. standard of care

A second critical point concerns the nature of the control group. In this study, the control group received no specific preventative intervention. However, the current landscape of post-miscarriage care is evolving. Major international guidelines, such as those from the AAGL and ESGE, recognize the role of post-operative estrogen therapy in promoting endometrial regeneration and preventing adhesion recurrence, considering it a standard of care in many clinical scenarios (3).

Comparing a novel “combined triple therapy” against a “no-treatment” arm creates a significant contrast that may not reflect real-world clinical dilemmas. The more relevant clinical question is not whether “something is better than nothing,” but whether ACP gel provides an incremental benefit when added to standard hormonal support. By utilizing a control group that received no hormonal support, the study potentially conflates the well-known benefit of estrogen with the specific efficacy of the ACP gel.

## Discussion

Sheng et al. (1) provided valuable data suggesting that a multimodal approach is both feasible and potentially beneficial. Given that the pathophysiology of IUA involves complex inflammatory and fibrotic pathways, it is biologically plausible that combining a mechanical barrier (gel) with hormonal endometrial support would yield superior results.

However, the leap from “biological plausibility” to “clinical evidence” requires rigorous control of bias. I strongly recommend that future studies on this topic adopt a randomized controlled trial design. If a retrospective design is unavoidable, statistical techniques such as propensity score matching should be employed to ensure that the control and intervention groups are balanced across all key prognostic factors.

In conclusion, while I commend the authors for addressing this challenging clinical entity, I urge clinicians to interpret the magnitude of the reported benefit with caution until it is validated by randomized trials that control for selection bias and utilize an active comparator group.
